# Previous life experiences and social relations affecting individuals wish for support when establishing healthy habits – a qualitative study of Norwegian Healthy Life Centre participants

**DOI:** 10.1186/s12889-021-11374-8

**Published:** 2021-07-05

**Authors:** Thea Ingebjørg Gjertsen, Anne-S. Helvik, Ingrid S. Følling

**Affiliations:** 1 Department of Clinical and Molecular Medicine, Faculty of Medicine and Health Sciences, Norwegian University of Science and Technology, Trondheim, Norway; 2 General Practice Research unit, Department of Public Health and Nursing, Faculty of Medicine and Health Sciences, Norwegian University of Science and Technology (NTNU), Trondheim, Norway; 3 Norwegian National Advisory Unit on Ageing and Health, Vestfold Health Trust, Tønsberg, Norway; 4 Centre for Obesity Research and innovation (ObeCe), St. Olavs University Hospital, Trondheim, Norway

**Keywords:** Healthy habits, Lifestyle, Qualitative research, Social relations, Sense of coherence, Allostatic load

## Abstract

**Background:**

Interventions to reduce and prevent overweight, obesity and T2D has been advocated worldwide. In Norway, Healthy Life Centres have been established to help individuals to reduce and prevent diseases, offering physical activity and dietary advice to establish healthy habits. Previous life experiences, social support and help from health personnel could play a role in the process of establishing healthy habits. The aim of this study was to explore how two groups of Healthy Life Centre participants described their previous life experiences, social relations and wish for support from Healthy Life Centre personnel.

**Methods:**

A qualitative design was used, including 49 individual semi-structured interviews. The interviews for this study were performed in two different samples, one sample of participants applying for HLC participation in 2013 (*n* = 23) and one sample of participants invited to HLC participation in 2015 (*n* = 26). The data was analyzed using systematic text condensation.

**Results:**

Three main themes in a chronological (past, present and future) order were identified: 1. Previous life experiences stamping life situation (past time). 2. Social relations being a support or a burden in everyday life (present time) and 3. Expressing wishes for HLC support (future).

**Conclusions:**

In the process of establishing healthy habits, the need for help from personnel may be differentiated based on previous life experiences and present social relations.

**Supplementary Information:**

The online version contains supplementary material available at 10.1186/s12889-021-11374-8.

## Background

Overweight, obesity and type 2 diabetes (T2D) have become major global health challenges [1, 2]. Interventions to reduce and prevent overweight, obesity and T2D should include diet advice, physical activity [3, 4] and behavioural therapy [5]. In some countries, there has been performed studies with interventions including dietary advice and physical activity aiming to reduce and prevent T2D in primary healthcare settings [5–7]. In Norway, Healthy Life Centres (HLC) have been established to support individuals to reduce and prevent disease by offering help to establish healthy habits [8], including offers within physical activity and dietary advice [8].

It is emphasized that individuals trying to change health habits may meet different barriers [9–11]. The barriers might include negative experiences from childhood, social life experiences [12] and family/work situation [10–12]. Adverse childhood experiences may increase the risk of unhealthy habits later in life [13]. Stressful life experiences during childhood can induce maladaptive coping responses and such experiences has been found associated with overweight and obesity in adulthood [14]. How individuals perceive life, and their degree of confidence and ability to handle life experiences, can be explained by allostasis and allostatic load [15]. Having too many stressors and challenges in life can induce allostatic overload, which predisposes individuals to disease [16]. One of the factors protecting against allostatic overload are social support [15]. Having positive relationships in the family, as well as good social network with great feeling of belonging, are important positive factors for establishing healthy habits [17]. A review found the role of social support from people’s natural environment to play an important role for establishing healthy habits [18] and support from family and friends may contribute to sustained change of behaviour [18–20]. Social network may provide support including information about diet or exercise, reassurance of steps necessary for healthy habits or by increasing compliance to treatment [18].

Individuals with less support and resources may have a need for more intensive follow-up from HLC personnel to help them establish healthy habits, compared to individuals with more resources [21]. Information, support and technical advice from health personnel may be a key factor in behaviour change [18]. However, a review have reported that social support from health personnel may have somewhat limited effect compared to support from individuals’ natural environment [18]. There are many factors influencing the effect of support from health personnel, and building a relation based on trust and respect between individuals and health personnel seems to be of importance to establish healthy habits [22–24]. HLC personnel are trained in use of motivational interviewing (MI) in conversation with participants to help them reflect about their situation and motivation to establish healthy habits [8], since the individuals’ own resources are important for establishing healthy habits.

It is recommended that the HLCs use the salutogenic philosophy as a theoretical framework [8]. The salutogenic approach is aiming towards gaining confidence in participants own coping skills and increase their ability to behavioral change. Salutogenesis explores what causes positive health outcomes and a life view which helps in achieving mastery. According to the salutogentic theory, the ability to use resources is termed as Sense of Coherence (SOC). SOC is a personal way of thinking, being and acting involving in inner tris, one which leads people to identify, benefit, use and re-use the resources at their disposal. The salutogenic philosophy includes reflection of life situations, a review of available resources and active adaptation to life stressors and challenges [25, 26]. Individuals aiming to establish healthy habits may have diverse life experiences and social support in everyday life [9–12, 15–20, 22]. Getting help from health personnel may be important in the process of establishing healthy habits [18, 21–24]. Such knowledge may contribute to improving HLC programs. The present study aimed to explore how two groups of HLC participants describe their life experiences, social relations and wish for support from HLC personnel.

## Methods

### Design

This study employed a qualitative design with individual semi-structured interviews of participants in two different situations, one group of participants applying for HLC participation in the initial phase of HLC programme in 2013 and one group of participants invited to HLC participation in an ongoing HLC programme in 2015. Given the exploratory nature of this study, a descriptive qualitative approach was most appropriate to elucidate insights in the participants’ life experiences, social relations and wish for support from HLC personnel.

### Participants

The inclusion criteria for the participants, applying for HLC participation and invited to HLC participation are presented in Table 1.

**Table 1:** Inclusion criteria

Participants applying for HLC participation (***n*** = 23)	Participants invited to HLC participation (***n*** = 26)
All new participants in initial phase of an HLC programme	Individuals identified with high risk for T2D in HUNT3 and accepted invitation to participate in an HLC programme as part of the VEND-RISK study
Age 18–70 years	Age 18–75 years
Men and women	Men and women
Being capable of giving informed consent to participate in the study	Being capable of giving informed consent to participate in the study

The group of participants applying for HLC participation were recruited from HLCs in two Norwegian municipalities: Levanger and Verdal. Eligible participants were asked by HLC personnel to be interviewed during an initial health conversation. The participants applying for HLC participation had not started attending any programmes at the HLC. The sample was strategic, based on age and gender. Recruitment continued until data saturation, resulting in 23 participants.

The group of participants invited to HLC participation took part in the Nord-Trøndelag Health Survey3 (HUNT3) and the VEND-RISK study, initiated by the Obesity Research and Innovation Centre (ObeCe) at St. Olav’s University Hospital, aiming to prevent T2D in people with obesity. Those invited to the VEND-RISK were identified with high risk for developing T2D in HUNT3 and were informed about the HLC programme aiming to establish healthy habits to prevent the development of T2D. Of the 322 individuals invited to the VEND-RISK study, 45 accepted to participate in the VEND-RISK with invitation to attend the HLC programmes from autumn 2013. Data from HUNT3 showed that for those 45 who accepted to participate in the VEND-RISK study, the socio-demographic, anthropometrics, diabetes risk, cardiovascular (CVD) and physical activity level measures were not different from the 287 non-participants. However, the women who participated in the VEND-RISK study differed significantly from those who declined with having more years of education and they were more employed compared to those who did not chose to participate. They also reported less that their health affected their social relations [27].

Out of these 45 participants who had been invited to attend the HLC programmes, an HLC nurse phoned asking them for being interviewed, preferably based on age and gender. Recruitment continued until data saturation was met. Altogether 26 participants were interviewed.

Thus, a total of 49 ethnic Norwegian participants were included, 23 participants applying for HLC participation and 26 participants invited to HLC participation respectively. Characteristics of the participants are described in Table 2**.**

**Table 2:** Characteristics of the participants

Characteristics	Participants applying for HLC participation (***n*** = 23)	Participants invited to HLC participation (***n*** = 26)
**Age**	18–29 (17%)	59–65 (16%)
	30–39 (17%)	65–69 (65%)
	40–49 (22%)	≥70 (19%)
	50–59 (22%)	
	> 60 (22%)	
**Sex**		
*Male*	7 (30%)	11 (42%)
*Female*	16 (70%)	15 (58%)
**Overweight (BMI > 25)**	19 (83%)	25 (96%)
**Support from Norwegian Labour and Welfare Administration**	13 (57%)	22 (85%) *

*16 were partially or fully retired

### Interviews

Interviews of participants applying for and invited to HLC participation were performed by the last author in two periods, February–April 2013 and February–March 2015. The interview guides were developed for two previous studies [12, 21]. A semi-structured interview guide with open-ended questions was used during the interviews (Additional file 1). Follow-up questions and probes were also asked. The main themes discussed in the interviews were regarding the participants’ health habits, their childhood and social resources, and their wish for help from the HLC personnel. The interviewer (last author) together with second author decided when data were saturated based on that no new information came up for the third time, i.e. from interviews 21–23 on the first group and from interviews 24–26 on the second group. For the second group it was considered saturation at interview 19–20, however, it came up new information again at interview 20 and then interviews were continued again. No new information came up after interview 26 and then saturation was met.

Interviews were audio recorded, and lasted between 14 and 78 min, with an average time of 35 min for the total sample. The mean duration for HLC participants applying for participation was 42 min and for those invited to HLC participation the mean duration was 28 min. The first 23 interviews (of those applying for HLC participation) were transcribed consecutively by last author. The next 26 interviews (of those invited to HLC participation) were transcribed consecutively after study inclusion, the 14 first by the last author and the 12 next by a professional typist.

In total the transcribed interviews, constituted of 628 pages whereas 234 pages from the 23 participants applying for HLC participation and 394 pages from the 26 participants invited to HLC participation.

### The HLC programme

The HLC offers a structured 12 week programme, both individually and group based, primarily with offering physical activity sessions, dietary courses and tobacco cessation courses [8]. HLC participants has the possibility to extend the period three times if necessary [8].

The HLCs offers mostly daytime group sessions, thus, once a week there is a session after ordinary work hours in the evening.

In addition to the standardized programmes at the HLC, the participants invited to HLC participation in the VEND-RISK study were offered to attend annual surveys, blood sample testing and physical activity tests for five years.

### Data analysis

The transcripts were imported into NVivo version 12 Pro, for coding and organizing the material. The material was analysed using systematic text condensation (STC) [28], in four steps [29]. In the first step, the first author listened to the audio files simultaneously as reading the interviews of the participants invited to HLC participation, to get an overall impression. Notes were written continuously, and a summary were made from each interview. Preliminary themes were written down. The same was done for the interviews of the participants applying for HLC participation, except audio files not being available. Second and last author read five interviews and the summaries from both participants applying for HLC participation and invited to HLC participation, and all three researchers met to discuss preliminary themes. In the second step, meaning units from the interviews were sorted into codes, followed by organizing codes into themes and subthemes. In the third step, the first author made a table with condensates from both applying and invited participants, divided into codes and subthemes. The table was made to compare participants applying for HLC participation with invited participants. The authors again met to discuss the table, themes, and subthemes. In the fourth and last step, the findings were summarized and written in full text. The quotations were translated from Norwegian to English. Throughout the process of analysis, the first author continuously checked the findings against transcriptions for validation. Being flexible in the process of analysis is a precondition to catch anything new in the material and following it up systematically [29].

## Results

**Fig. 1:**
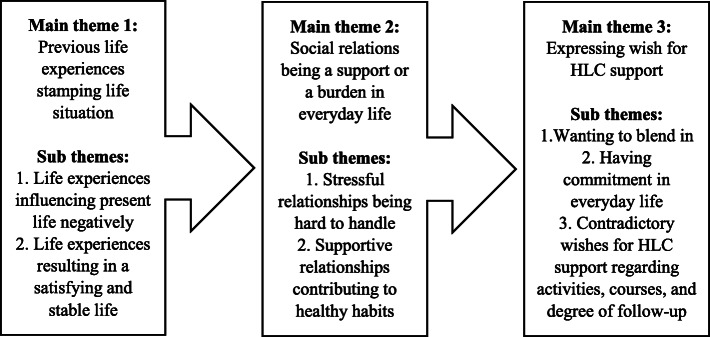
Main themes and sub themes

From the HLC participants’ stories three main themes emerged, each containing 2–3 sub themes (see Fig. 1). The themes are presented in a chronological order (past, present and future) for how they described life experiences, social relations and wish for support from HLC personnel. Quotes illustrating the themes and sub themes are presented in Table 3.

**Table 3:** Quotes from interviews

Main theme 1 Previous life experiences stamping life situation	
*Participants applying for HLC participation*	
Sub theme 1: Negative life experiences influencing present life “I have tried losing weight different ways (…) I have PCOS, which makes it hard to get pregnant. So I lost 34 kg and got pregnant, and then I regained the weight (..) I did a gastric bypass operation and lost fifty-two kg, and cannot find myself afterwards. I quitted work when I got children because I wanted to stay home with them. Now I have NAV contacting me regularly, telling me I need to get a job because I am unemployed …” Woman, age 35–44	
Sub theme 2: Positive life experiences resulting in a satisfying and stable life “I have lived in the same town all my life. I worked at the same place for 30 years.” Man, age 55–64	
*Participants invited to HLC participation*	
Sub theme 1: Previous negative life experiences influencing participants life *“My childhood was difficult. I got a lot of beating. I was the oldest one … In addition, my mother got beaten, and I saw that as a child. I remember it very well. I have been through a lot in my life … I have been in a lot of pain, but apart from that, I feel rather fine today.”* *Man, age 65–75*	
Sub theme 2: Positive life experiences resulting in a satisfying and stable life “*… we were lucky since my father worked for the government, and our economy was alright. I grew up in a small community, where all were family, and we did not even lock our doors to the house. My childhood was safe and nice. And as an adult I have lived quite normal, I would say.”Woman, age 65–75*	
**Main theme 2 Positive life experiences resulting in a satisfying and stable life**	
*Participants applying for HLC participation*	
Sub theme 1: Stressful relationships being hard to handle *“I live by myself with my youngest daughter aged fifteen. (…) She struggles with psychiatric problems, just like me. We are not that much outside meeting other people.”* *Woman, age 45–54*	
Sub theme 2: Supportive relations contributing to healthy habits *“You must have support by your family, it is important, if not I don’t think I would get further on. The kids are also very engaged in eating healthy, and they can see I am getting into better shape, and maybe happier as well, when I get healthy food and get the one hour walk during the evening as well.”* *Woman, age 35–44*	
*Participants invited to HLC participation*	
Sub theme 2: Supportive relationships contributing to healthy habits *“Getting out of the house is incredibly hard sometimes, and for some people it is impossible. You need a boost, and you need someone who can knock on your door and tell you: Let us go for a walk. It is a pleasure having the opportunity to go for a walk together with another person. It certainly is.”* *Woman, age 55–64*	
**Main theme 3 Expressing wishes for HLC support**	
*Participants applying for HLC*	
Sub theme 1: Wanting to blend in to blend in *"The size of the groups has not been that big, which I think is important. No one has been better than others have. We have been on the same level. We have all been similar. (…) The groups have been very nice."* *Woman, age 45–54,*	
Sub theme 2: Having commitment in everyday life *“I know that when I am done with the program at the HLC I have to find other things to commit to …”* *Woman, age 45–54*	
Sub theme 3: C Contradictory wishes for HLC support regarding activities courses and degree of follow-up *"I participate in most of the courses, food course, football, indoor cycling, strength training and motivational groups. And conversations with the physical therapist."* *Woman, age 18–24*	
*Participants invited to HCL*	
Sub theme 1: Having a need to blend in *“… I did not know if this was the right place for me, because I felt very healthy compared to many others”* *Woman, age 55–64*	
Sub theme 2: Having commitment in everyday life *“Every Monday I meet a couple of ladies for an hour, and we work out together. We have done it for many years (…) And during summer we bike.”* *Woman, age 65–75*	
Sub theme 3: Contradictory wishes for HLC support regarding activities courses and degree of follow-up *“I have not participated in any of the courses or activities at the healthy life center. I have other activities I go to and are happy with those.”* *Man, age 65–75*	

### Main theme 1: previous life experiences stamping life situation

#### Sub theme 1: life experiences influencing present life negatively

Most of the participants applying for HLC participation had life experiences influencing their lives negatively, experiences which they still struggled with and were affected by. Several participants described their lives as “quite turbulent”. They were struggling with little confidence as adults, because of difficult relational experiences previously in life. One participant described a childhood with violence. As an adult, she had psychological problems and suicidal thoughts. Participants said they repeatedly had been bullied by close family members or friends as children or adolescents which inflicted their present confidence negatively. Others described violence done by previous partners and death threats that contributed to several struggles nowadays, especially with mental health.

Of the participants invited to HLC participation, only one described difficult life experiences concerning family violence, accidents and loss of family members in the past. Despite these experiences, he said he no longer was affected by them and stated that he felt “rather fine today”.

Participants applying for HLC participation commonly described having experienced different losses, including family members and spouses. One mentioned many close relations died the last years: both parents, an uncle, and friends of the family. The loss of these relations was hard and still controlled her life. In addition to these life experiences, participants applying for HLC participation described being troubled with a poor health throughout life. Some of the health issues the participants had were depression, schizophrenia, and problems with alcohol. Multiple had several sick leave periods from work or were out of work, because of health issues, and description of previous negative experiences with health care institutions and professionals, not receiving the support they experienced needed were common. Commonly they throughout life had trouble with either keeping work, fulfilling their work obligations, being bullied at work, or being told not to be good enough at work.

#### Sub theme 2: life experiences resulting in a satisfying and stable life

Among the participants invited to HLC participation, the majority described life experiences resulting in a good and stable life. They told about a safe and nice childhood, good social relations and multiple participants had been living in the same area their whole life. As adults, the participants had experienced stability in their work situation. Contrary to participants invited to HLC participation, it was rare for participants applying for HLC participation to point to previous life experiences creating good memories, with a few exceptions.

Overall, the participants invited to HLC participation described growing up with healthy traditional Norwegian food and had an active everyday life. As adults, they were aiming for knowledge to keep healthy, and one participant said he had participated in a course about diabetes at the hospital to get more knowledge about the disease. Participants invited to HLC participation commonly said they were involved in voluntary organizations throughout their adulthood, which enriched their life, also today. They described their health as good and that they stayed mostly healthy. Some participants had chronic diseases and were satisfied with the help they received from the health care system and highlighted having a good relationship with their GP.

### Main theme 2: social relations being a support or a burden in everyday life

#### Sub theme 1: stressful relationships being hard to handle

Most of the participants applying for HLC participation described at present struggling with stressful relationships within families and/or social life and experienced these relations as a burden. Some participants had divorced from a difficult marriage, but still described it as hard to handle the new life situation. Other participants mentioned not getting support by their spouses to establish healthy habits, exemplified by spouse not wanting to be physically active together with the participant because they were in a too bad shape, or by spouse eating unhealthy because he did not gain weight.

*My partner is not physically active together with me, because he thinks I walk too slowly, even though I walk so fast that I feel like I am going to die. So … I walk mostly by myself.*

*Woman, age 45–54, participant applying for HLC participation.*

A common description among the participants applying for HLC participation was taking care of all others but themselves, with few people to support them. For example, one participant pointed out that she was the only relative of her parents, and with her mum having dementia and living with her father, she had to help and support them both substantially. The participants expressed a small network, and when they had problems with their closest relations, they had few other relations giving them support.

Remarkably and contrary to the participants applying for HLC participation, none of the participants invited to HLC participation described any negative or demanding relations in their present life.

#### Sub theme 2: supportive relationships contributing to healthy habits

A few of the participants applying for HLC participation described experienced support from their close family, which made them feel seen and understood. The ones who had support by their families described the support as a motivation to change. Establishing healthy habits and new habits could become a family project. One said “my partner makes the same changes as me. He does everything to help me”. They also described their family as their network. However, only a minority of the participants applying for HLC participation expressed having friends or other relations being supportive in everyday life.

The participants invited to HLC participation generally experienced a positive and well-functioning relationship with their close family supporting healthy habits. Most of them had a large part of their family (parents or offspring) nearby, and often met them in their daily life. They mutually respected and supported each other. The whole family had the same interests, including being active and having healthy habits. One participant described the relation to the spouse by saying that “when you are two people living together, you can do changes together if it is necessary for the partner”. In most cases, the participants invited to HLC participation highlighted a great and well-functioning social network also outside the family. They were busy with different projects and participating in volunteer organizations, and thus, they had regular appointments and organized activities, meeting friends and associates. Many of them told about participation in group activities for years. The value of social relations to become and stay physically active was emphasized.

Some participants invited to HLC participation even said there could be too many social happenings during the week. Nevertheless, they were satisfied with the social network they were a part of, and one participant stated he had a “perfect network”:

*As retired, I try to be active very day. I participate in something called “Senior Forum” as a secretary, and my partner says I spend 50% working hours on it. (…*) *We have a lot of visitors and we visit others a lot, I never feel lonely. (…) I have a perfect network around me. And I think it is fun because I have two grandchildren who need a bit help with homework, which I think is very enjoyable.*

*Man, age 65–75, participant invited to HLC participation.*

### Main theme 3: expressing wish for HLC support

#### Sub theme 1: wanting to blend in

The participants applying for HLC participation highlighted meeting others in the same situation as a positive factor when attending the HLC. Having the possibility to be physically active together with others as an advantage. Having respect and inspire each other, as well as motivate each other were important factors for the participants. The size of the groups they participated in were described as good, with “no more than ten participants to benefit from the group”. The group members had time for each other, they were on the same level, the participants felt safe and the atmosphere was good and wanted to keep in touch with other participants when they were done at the HLC. It was also highlighted by the participants to be positive that they met others in the same situation.

*“It is nice to get out and be social and meet similar people, instead of just hiding inside. Woman, age 35–44, participant applying for HLC participation.*

The participants invited to HLC participation on the other hand, expressed a feeling of not blending in the groups at the HLC. Some men mentioned that there were mainly women in the groups, while a woman had experienced being asked stupid questions repeatedly. The spread in physical shape in the groups was too broad, and the other group members at the regular HLC had different health problem from them and having a worse health condition. The feeling of being too healthy was mentioned. Participants emphasized that to benefit from being in a group, the group needed to have something in common.

#### Sub theme 2: having commitment in everyday life

Participants applying for HLC participation described having an appointment as an important factor for establishing healthy habits. Having a commitment gave the participants motivation to get out of the house, and it helped them to start establishing habits. One participant mentioned she had many negative thoughts when just being home, but when attending the HLC her mood got better. Participants described habits like being passive, doing nothing during the week and just being inside the house. The HLC was a good place to go to, and a great bonus that it was without financial cost. Going to the HLC regularly helped participants structuring the day and being active.

Participants invited to HLC participation described being active, having many activities from earlier, and not having time for the activities at the HLC. One participant said she wanted to participate in the dietary course, but she was always busy. However, it was expressed that the activities at the HLC were “a very good offer for people who do not have other activities”*.* Multiple participants said they regularly went to a fitness centre and were able to eat healthy and work out on their own.

*They had activities at two different places (…*). *But it was not necessary for me, because I already went to training at the local gym every day.*

*Woman, age 55–64, participant invited to HLC participation.*

#### Sub theme 3: contradictory wishes for HLC support regarding activities, courses, and degree of follow-up

Participants invited to HLC participation highlighted the annual follow-up meeting and testing in the VEND-RISK study. They experienced these meetings and tests as helpful to maintain healthy habits. The participants invited to HLC participation decided to not participate in any of the regular HLC activities offered them, and the decline was reasoned in lack of time, need and interest. Thus, they talked about a wish to obtain and maintain healthy habits to live their life with a wish for a long life with good health as a future perspective. Having regular conversations with the personnel at the HLC were a good reminder, and participants were able to keep in shape themselves. Getting feedback was important, and having tests and conversations were a good way to keep track on their health status.

Participants applying for HLC participation highlighted that they wanted to join all the activities and courses available. The programme at the HLC was described as very good. Participants also emphasized the possibility to exchange experiences with others. One participant said the days became brighter after she started at the HLC. Multiple participants described a huge benefit in participating in the different courses, and the HLC had contributed to great changes in their life. They also talked about that they would need a long period of follow-up and that 12 weeks was a short time. They also wanted to meet co-participants and they talked about a wish for having social meeting points like walking groups after the period at the HLC.

In addition, the participants applying for HLC participation expressed a need for someone to talk with and to get support while participating in the programme.

At the HLC the participants experienced the health personnel to be interested, not judging, and meeting them in their life situation. Participants got individual guidance, and reassured when working out, while realizing that “the body tolerated to be used”. In addition, the participants applying for HLC participation said HLC pushed them to reach their goals, as well as participants were taken seriously. One participant said it was important for her that she felt she meant something for the health personnel, and that her needs and wishes were seen, because this was missing in her life.

*… it feels safe and nice, because I have one person here who follows me and really wants me well and want everything to be fine. We have conversations (…) and she sees me as a whole person. (…) She is really seeing me.*

*Woman, age 25–35, participant applying for HLC participation.*

## Discussion

This study identified three main themes in a chronological order: 1. Life experiences stamping life situation (past time). 2. Social relations being a support or a burden in everyday life (present time) and 3. Expressing wishes for HLC support (future). Participants’ previous life experiences and present social situation affected the wishes for support from the HLC. Life experiences influencing present life negatively, and stressful social relations made it difficult to establish healthy habits, resulting in an increased wish for support from HLC personnel. In contrast, life experiences contributing to a stable and satisfying life, and supportive social relations resulting in a restricted wish for HLC support. Thus, the expressed wish for HLC support seemed to differ for the participants applying for and invited to HLC participation, based on previous life experiences and present social situation.

### A connection between previous life experiences, social relations, and a wish for support

This study revealed that most of the participants applying for HLC participation had life experiences influencing their present life negatively. In contrast, most of the participants invited to HLC participation described life experiences resulting in a satisfying and stable life.

Other studies have found that previous difficult life experiences may increase the risk for allostatic load [15] and unhealthy habits later in life [13, 14], as well as being barriers for establishing healthy habits [9–11]. Life experiences in childhood and young life influence grow of SOC in adult life [30]. A high allostatic load due to previous negative experiences and poor health may influence their way of thinking and ability to use and re-use their inner resources. A study found individuals having low SOC and poor perceived health, had little social and emotional support [31]. Those having a high SOC may cope with stressful situations and stay healthy in a more advantageous way than individuals with low SOC [32], and thus, avoid allostatic overload. However, the SOC of the participants in the present study is unknown. Even so, when having experienced challenging life experiences, like the participants applying for HLC participation described, it is likely that SOC are lower than in those with less stressful previous life experience. Thus, establishing healthy habits without assistance from social network or professionals are made harder. A challenge for HLCs personnel could be to help participants with fewer coping resources and give them the support and follow-up necessary for them to establish healthy habits. In such cases HLC personnel could get assistance from and collaboration with other municipal services, e.g. psychologists.

Furthermore, the participants applying for HLC participation had stressful relationships within families and/or social life, whilst most participants invited to HLC participation had close relations with family and friends and received social support in everyday life. Other studies have shown that social support is an important coping resource for achieving and maintaining health behaviour change [18, 20]. Also, individuals receiving social support may be better able to cope with negative stressful experiences [33]. Frequent and substantial stress may promote allostatic overload [34]. For participants applying for HLC participation, their stressful relationships with families and/or restricted social life, as well as their previous difficult experiences in life still affecting them, might together have resulted in a high allostatic load and low SOC. Thus, they are being in need for more support from health personnel when establishing healthy habits than those with life experiences resulting in a satisfying and stable life and close relations with family and friends and received social support in everyday life. Even so, it is not always that people with negative experiences and little social resources have issues regarding health habits, like overweight and obesity [14], and thus, need for more support.

### Receiving support from health professionals or family/friends

Both the participants applying for and invited to HLC participation described having social support as important when establishing healthy habits. However, the social network from family and friends for such support differed considerably between the groups of participants. The participants applying for HLC participation with stressful relationships, also hard to handle, expressed comprehensive needs for support from HLC personnel. These HLC users may experience that they had limited resources to their disposal, which reduced their mastery, and thus, they sought comprehensive support from HLC personell. This is in line with the Salutogenic philosophy [25, 26].

It is repeatedly found that support is important in order to establish new and healthy habits [17, 35]. Even so, participants seeking HLC programmes may not be able to establish healthy habits without support from both family/friends and health personnel [24]. HLC personnel aims to listen and see participants without prejudice [22], and a trustful and respectful relationship between participants and HLC personnel has been found important [22–24]. HLC personnel focuses on empathy and respect for participants self-determination and own choices, and use MI as a conversation tool with participants [8]. The aim of utilization of MI is always behavioral change. However, we do not know much about the quality of MI performed, and we cannot rule out the possibility of an empathic approach to a degree that undermines behavioral change. Nevertheless, even though support from health personnel may be a key factor in behaviour change, but the support may have somewhat limited effect compared to support from peoples’ natural support network [18]. Thus, since changing behaviour mostly happens in peoples’ private and work setting [18], it might be advantageous to explore which participants at HLCs who are in need of strengthening their social network and/or need more support from HLC personnel. Staying in touch with other participants at the HLC might be a way of expanding participants’ network and social support, and HLC personnel could focus on organizing this as a part of the intervention.

### Strengths and limitations of the study

More women than men are participating at HLCs [36] and this study also included more women than men. Thus, this study might therefore have a gender representative composition of participants in HLC programmes. In this study with two groups of participants, the group of participants invited to HLC participation had no participants younger than 59 years, while the youngest in the group of participants applying for HLC participation was 18 years. This might bias the results since increasing age is linked to experience social relationships more satisfying and positive [37].

In this study, 49 participants were interviewed creating a great amount of information and a rich material, but may also lead to superficial analysis [29]. Nevertheless, the large data variations, enables illustration of different aspects of the research question, which is important in qualitative research [29]. Even though we had a high amount of information, the background information was somewhat limited and more information regarding background could possibly have enriched the understanding of experiences and social relations in the two groups of participants, but the presented information should be adequately to help readers to understand and interpret the results of this study. For the study rigour, the design was to elicit descriptions of the participants life world in order to interpret the meaning which the phenomena they described held for them. The interviewer chose to follow the themes the participants raised as being important. The interview guide was intended to assure that the interviews would remain open-minded, without the questions being standardized. For the study trustworthiness, in the interview setting, a friendly and empathetic approach was emphasized and the interviewer aimed to build trust to ensure equitable balance of power. No attempt was made to manipulate the conversations toward any particular phenomena of interest and unexpected topics arouse during the interviews regarding personal stories for the participants applying for HLC programmes. The same process was followed for the interviews with participants invited to the HLC programmes, though the interviews were not as personal as the first group and more of those participants were speaking more often about positive feelings.

The first author, main responsible for the analysis, did not collect the data, and thus some information may have gotten lost in the transcription [29] [38]. Even so, the last author who performed the interviews took part in the analysis group and the analytic steps together with the second author. These actions may have prevented information getting lost. Also, an analysis group may give attention to more details, and is seen as an advantage [29].

The data for this study was collected five-seven years ago. The organization of the HLCs programmes in the Norway has not changed over the last ten years since the first National Guidance for the establishment and organization of HLCs from the Norwegian Directorate of Health was published. Furthermore, this study provides insight about participants life experiences, social relations and wish for support from HLC personnel that could be helpful for how HLC programmes are delivered regardless of time since data collection. With the cross-sectional nature of the present study, we do not know if their wish for support for HLC to establish and maintain healthy habits were fulfilled, and we do not know how they cope without HLC support. This perspective could preferably be followed-up in a new study of participants after their HLC intervention and follow-up ended. After ten years, it remains to be seen who actually can benefit from HLC intervention programmes, and who will need other approaches to lifestyle changes, such as social or psychological interventions, or specialized health care treatment. There is a need to evaluate if the HLC fulfilling their role to promote public health with the resources and organization available. Future studies should examine how lifestyle interventions in the Norwegian primary health care system are organized and how personnel can help participants with and promote lasting lifestyle changes.

## Conclusions

In this study, 49 HLC participants highlighted three main themes, representing participants’ past, present, and future when talking about establishing healthy habits: previous life experiences, present social relations, and the wish for support from the HLC personnel. The participants applying for and invited to HLC participation had different previous life experiences and present social situations that somehow resulted in contradictory wishes for HLC support. Stressful relationships being hard to handle as well as previous life experiences influenced the participants applying for HLC participation present life negatively, which increased their wishes for HLC support. In contrast, the participants invited to HLC participation had life experiences with a stable, satisfying life, and supportive relationships contributed to healthier habits, resulted in less comprehensive wish for HLC support.

## Supplementary Information

**Additional file 1.** Interview guides. Description of interview guides of participants applying for and invited to HLC participation

## Data Availability

The raw data supporting the findings in this article can be found at the Centre for Obesity Research and Innovation. Due to the Regional Committee for Medical and Health Research Ethics in Central Norway regulations, we must secure the anonymity of the participants. In the raw data it is possible to identify the participants, and restrictions therefore apply to the availability of these data.

## Abbreviations

GP: General practitioner
HLC: Healthy Life Centre
HUNT3: The Nord-Trøndelag health study 3
MI: Motivational interviewing
REK: The Regional Committee for Medical and Health Research Ethics in Central Norway.
SOC: Sense of Coherence
STC: Systematic text condensation
T2D: Type 2 diabetes
VEND-RISK: The lifestyle intervention programme in two municipalities in Nord-Trøndelag
