# Supplementary material for: Previous life experiences and social relations affecting individuals wish for support when establishing healthy habits – a qualitative study of Norwegian Healthy Life Centre participants
Source: BMC Public Health. 2021 Jul 5;21:1315. doi: 10.1186/s12889-021-11374-8 (PMC8256571; doi:10.1186/s12889-021-11374-8)
Supplement: Supplementary file 1 — Additional file 1. Interview guides. Description of interview guides of participants applying for and invited to HLC participation [file 12889_2021_11374_MOESM1_ESM.docx]

**Interview guides**

Interview guides of participants applying for HLC participation and participants invited to HLC participation were made for two previous studies but have not been published elsewhere. However, main themes are described in this document.

**Participants applying for HLC participation**

Main interview questions were: *“Could you tell me about your thoughts before attending the programmes at the HLC?”, “What expectations do you have to yourself when you are going to start at the HLC?”, “What expectations do you have to the other participants?”, “What expectations do you have to the personnel working at the HLC?”, “What expectations do you have to the programmes?”, “How do you think a period at a HLC can help you to change health behaviour? and “How do you think it is going to be to continue afterwards with the changes when you are on your own?”* (1).

**Participants invited to HLC participation**

Main interview questions were: “*How has your health and lifestyle been through your life?*”, “*How did you react to the information about being at risk for type 2 diabetes?”* and *“How has the VEND-RISK intervention programme influenced your lifestyle?”* (2). The interviews proceeded as a conversation, with follow-up questions *“Did you do any changes based on the knowledge about your risk?” and “What experience do you have with changing habits in diet and exercise?”*, with the goal of exploring what informants considered to be important.

**References**

1. Følling IS, Solbjør M, Helvik A-S. Previous experiences and emotional baggage as barriers to lifestyle change - a qualitative study of Norwegian Healthy Life Centre participants. BMC Fam Pract. 2015;16(1):73.
2. Følling IS, Solbjør M, Midthjell K, Kulseng B, Helvik A-S. Exploring lifestyle and risk in preventing type 2 diabetes-a nested qualitative study of older participants in a lifestyle intervention program (VEND-RISK). BMC Public Health. 2016;16(1):876.
